# Production of a risk mitigation matrix and four-grade threshold scale using Cox Regression to predict osteoarthritis development

**DOI:** 10.1515/biol-2025-1193

**Published:** 2026-01-13

**Authors:** Laura Jane Coleman, John L. Byrne, Stuart Edwards, Rosemary O’Hara

**Affiliations:** South East Technological University, Kilkenny Road Campus, Kilkenny Road, Carlow, Ireland; UPMC Aut Even Hospital, Freshford Road, Kilkenny, Ireland

**Keywords:** survival analysis, inflammatory biomarkers, risk classification, predictive diagnostics, translational medicine

## Abstract

Prognostic markers have become essential in predicting disease progression, particularly in osteoarthritis (OA), where early detection can improve clinical outcomes. This study applied survival analysis, specifically the Cox proportional hazards model, to evaluate the role of biomarkers (IL-6, TNF-α, and MPO) in OA progression. A risk mitigation matrix was developed to stratify individuals based on Xbeta values obtained through Cox Regression, allowing for an evidence-based approach to risk classification. A four-grade threshold scale was constructed to refine diagnostic precision by categorising risk into four levels. The study found that IL-6 concentrations ≥ 6.95 pg/mL, TNF-α ≥ 40.51 pg/mL, and MPO ≥ 5.45 pg/mL were indicative of early-stage OA, while hazard thresholds marked advanced disease risk. The risk mitigation matrix aligned with these findings, demonstrating its applicability in clinical decision-making. Integrating Cox Regression with Discriminant Function Analysis (DFA) improved validation, ensuring robust risk stratification. These findings contributed to advancing OA diagnostics by providing quantitative thresholds for early intervention.

## 1 Introduction

Osteoarthritis (OA) is a degenerative joint disease that significantly impacts quality of life and functional ability, particularly in older adults [1], [2], [3]. It is characterised by progressive cartilage degradation, joint space narrowing, and subchondral bone remodelling, leading to pain, stiffness, and reduced mobility [4]. Despite its high prevalence, early detection of OA remains a challenge, as conventional diagnostic methods such as radiography and MRI primarily detect OA in later stages [5], highlighting the need for reliable prognostic biomarkers to enhance patient outcomes by improving risk stratification and guiding early therapeutic decisions [6].

Prognostic markers, including Interleukin-6 (IL-6), Tumour necrosis factor alpha (TNF-α), and Myeloperoxidase (MPO), have been identified as potential indicators of disease progression due to their roles in systemic inflammation and oxidative stress [7]. IL-6 and TNF-α are key cytokines mediating synovial inflammation and cartilage degradation, while MPO, reflects neutrophil activity and oxidative tissue damage [8], 9].

However, these biomarkers are not without complexity. IL-6 exhibits context-dependent actions, functioning as both a pro- and anti-inflammatory mediator [10], [11], [12], [13], and although it correlates with radiographic severity in many studies [14], [15], [16], [17], [18], [19], findings remain heterogeneous. TNF-α, while strongly linked to cartilage degradation and synovitis [15], [20], [21], [22], [23], [24], is also elevated in systemic conditions such as prostate cancer and age-related mortality [25], 26], limiting its specificity. Similarly, MPO levels are elevated in early OA but decline in advanced disease despite ongoing inflammation [27], 28], with expression also reported in osteophytes [29] and in other inflammatory diseases such as psoriatic arthritis [30]. Such variability across disease stages, biological matrices, and co-morbid conditions underpins the inconsistent results reported in previous studies and necessitates robust modelling approaches to validate their prognostic value.

Blood based assays of these markers are clinically attractive due to being minimally invasive compared with synovial fluid or tissue sampling, which although more specific, are less practical for routine monitoring [31], 32]. Their utility lies in the ability to stratify fast progressing patients, guide therapeutic decision making and optimise clinical trial design [33]. Therapies such as IL-1inhibitors, and inverse agonists of retinoic acid-related orphan receptor alpha (RORα) antagonists, and cartilage-anabolic agents like fibroblast growth factor-18 (FGF-18) and bone morphogenetic protein-7 (BMP-7) intersect with inflammatory and oxidative stress pathways, suggesting that IL-6, TNF-α, and MPO may also function as surrogate markers for monitoring treatment response [34], 35]. IL-6 signals via the janus kinase/signal transducer and activator of transcription (JAK/STAT) pathway [35], 36], TNF-α activates the nuclear factor kappa-light-chain-enhancer of activated B cells (NF-κB) and mediated by mitogen-activated protein kinase (MAPK) cascades [37], and MPO drives neutrophil-mediated oxidative damage [27].

However, their broader clinical applicability has been limited by variability in assay methodologies, where detection rates can be as low as 30–50 % despite higher specificity [18], 38], inconsistent hazard ratio estimates and small sample sizes [39]. Furthermore, differences between serum and plasma profiles must be considered, as TNF-α and MPO in particular have shown variability across matrices, reinforcing their value as diagnostic and prognostic markers of OA [40], [41], [42], [43], [44]. These challenges highlighted the importance of applying robust statistical frameworks to validate biomarker thresholds in OA. Importantly, biomarkers could be tracked longitudinally to distinguish between normal biological activity, pathological change, and therapeutic response [45].

This study employed Cox Regression analysis to refine biomarker-based OA risk stratification, a four-grade threshold scale and a risk mitigation matrix was constructed to enhance early diagnosis and intervention strategies. Previous studies have demonstrated the applicability of the Cox proportional hazards model in OA research. Cox proportional hazards model first introduced in 1972, is a foundational tool in survival analysis, designed to evaluate the relationship between covariates and time-to-event outcomes in the presence of censoring [46]. Its semi-parametric structure allows hazard estimation without assuming a specific baseline hazard function, making it highly flexible and widely applicable [47]. Survival analysis is particularly suited to chronic disease research, where individuals may not have developed symptoms during the study period [48]. In OA research, for example, Xu et al. linked dietary factors to knee OA progression using Cox Regression, accounting for demographic, socioeconomic, and clinical covariates [49].

Studies have expanded the utility of Cox models, particularly in handling non-proportional hazards and complex observational designs. Weighted Cox Regression was proposed for estimating average hazard ratios in the presence of time-dependent effects enabling more interpretable effect sizes [50]. Robust estimation through trimming was also introduced, improving reliability in datasets with outliers or extreme values by reducing their undue influence on model fitting [51]. Cox Regression was further extended through the integration of sampling weights into robust estimation procedures, addressing challenges commonly faced in case-cohort studies and enhancing model validity in stratified populations [52].

These methodological adaptations are particularly relevant in the context of OA, where small sample sizes, inter-patient variability, and the presence of biomarker outliers pose significant analytical challenges. Traditional methods often struggle to capture the heterogenous and non-linear progression of OA. To address this, the present study adapted Cox Regression to use biomarker concentration data as a surrogate for time-to-event modelling. While not the conventional application, this novel approach allowed the derivation of survival-like and hazard thresholds. This approach aligned with other innovations in survival analysis and allowed for the development of biomarker-based risk classification framework tailored to OA progression [50], [51], [52].

Despite these advances, challenges persist in OA prognostic research. Many studies fail to report key statistical metrics, with variability in study methodologies, inconsistent reporting of hazard ratios, and limited sample sizes [39]. Furthermore, inconsistencies in cut-off levels for biomarker thresholds make it difficult to establish standardised diagnostic criteria. Riley emphasised the advantages of individual patient data analysis, which enables greater flexibility in defining marker combinations, adjusting cut-offs, and improving risk stratification accuracy [53]. More recent studies have highlighted the importance of considering both probability and impact in risk assessment, which aligns with our approach to developing a risk mitigation matrix [54], 55].

While biomarker concentrations serve as primary predictors, lifestyle factors such as BMI, diet, physical activity, and cholesterol levels were not included in this analysis due to ethical and logistical constraints. Although this may be seen as a limitation, focusing solely on biomarker-based risk prediction allows for a more direct evaluation of inflammatory and oxidative stress markers in OA progression. Future research incorporating larger sample size, multi-centre collaborations and additional patient variables would be necessary to enhance the generalisability of these findings [56].

To address these limitations, this study developed a biomarker-based threshold system for OA prognosis, integrating Cox Regression-derived Xbeta values into a risk mitigation matrix. This approach enabled the derivation and validation of quantitative cut-off values for IL-6, TNF-α, and MPO, allowing stratification of four clinically meaningful risk categories. Risk mitigation matrices provided an additional layer of analysis, incorporating both clinical and serological markers to estimate disease progression risk and inform treatment decisions [57]. These matrices, widely used in epidemiological research, have been applied in studies ranging from cardiovascular disease to infectious disease modeling [58], 59]. However, their application in OA research remained limited. By combining Cox Regression with a risk mitigation matrix and DFA validation, the study not only identified biomarker thresholds predictive of OA progression but also embedded them within a structured framework. The four-stage system developed here provided a foundation for a clinical decision support tool, advancing personalised prognosis and informing treatment strategies in OA management. In doing so, this work bridges exploratory marker research with clinical application offering a reproducible model to improve the accuracy and utility of prognostic assessment in OA.

## 2 Methods

### 2.1 Study design and ethical approval

This study is a prospective observational cohort study, classified as Level IV evidence. It was conducted in accordance with the Declaration of Helsinki and approved by the Ethics Committee of South East Technological University, Carlow (Protocol Code 160, approval date: 8 December 2016).

**Informed consent:** Informed consent has been obtained from all individuals included in this study.

**Ethical approval:** The research related to human use has been complied with all the relevant national regulations, institutional policies and in accordance with the tenets of the Helsinki Declaration, and has been approved by the Ethics Committee of South East Technological University, Carlow (Protocol Code 160).

### 2.2 Study participants and data collection

This study included 58 patients with severe knee or hip OA (mean age 71.7 ± 8.3 years) undergoing joint replacement surgery, and 28 healthy volunteers (mean age 32.0 ± 11.0 years). OA patients were diagnosed radiographically according to Kellgren–Lawrence grades 3–4, reflecting severe joint space narrowing, osteophyte formation, and bone deformity [60], [61], [62]. Volunteers were asymptomatic donors with no clinical or radiographic OA. Exclusion criteria included autoimmune or inflammatory disease, active infection, or current immunosuppressive therapy.

Previous analyses have shown that gender had no significant effect on biomarker levels, while age has only a partial influence, with TNF-α and MPO remaining significant predictors after adjustment [63]. These findings indicated that demographic variability did not substantially alter biomarker associations in severe OA. Consequently, detailed epidemiological stratification was not the primary focus in this exploratory study, which instead aimed to establish biomarker thresholds for risk stratification. Future longitudinal studies in larger, more diverse cohorts would be required to confirm these associations and to integrate demographic variables more comprehensively into predictive models.

### 2.3 Data preparation

Linear interpolation was applied to address missing data points within the dataset, estimating unknown values between adjacent observations [64]. To account for the non-normal distribution, log_10_ transformation was performed_._ All biomarker data was then standardised using z-score normalisation, which expressed values as deviations from the mean in standard deviation units [65]. These steps reduced bias, enabled comparability across biomarkers, and ensured appropriate scaling for Cox Regression analysis. Data availability details are provided in the Ethical and Legal Declarations Template.

Plasma and serum samples were obtained from both patients and volunteers to enable cross-matrix comparison and to account for potential variability between sample types. Biomarker concentrations of IL-6, TNF-α, and MPO were measured using commercially available enzyme-linked immunosorbent assay (ELISA) kits (Biolegend, London, UK), according to manufacturer’s instructions. All assays were performed in duplicate, and mean values were used for analysis. Calibration curves were generated using kit standards, with R^2^ values consistently >0.99 to ensure assay reliability. The samples were collected in the morning to minimise diurnal variations and were stored at −80 °C until analysis to prevent degradation.

Assay performance was monitored with intra-assay coefficients of variation (CV) < 10 % for all biomarkers. Concentrations falling below the lower limit of quantification (LLOQ) or above the upper limit of quantification (ULOQ) were excluded rather than imputed, with fewer than 5 % of data points affected. These steps ensured that only quantifiable and reliable values were incorporated into Cox Regression modelling.

### 2.4 Cox Regression analysis

Cox Regression analysis was carried out using SPSS^®^ version 25 to evaluate the relationship between biomarker concentrations and osteoarthritis risk. Traditionally, the Cox proportional hazards model estimates the effect of covariates on the hazard (risk) of a specified event occurring over time, using the semi-parametric model first proposed in 1972 [46]. In this study, the model was adapted to treat biomarker concentrations as the primary covariates of interest, with disease status used to simulate a survival-like endpoint. The model takes the general form:

$$h\left(t\right)=h_{0}\left(t\right)\;x\;\exp\;\left(b_{1}x_{1}+b_{2}x_{2}+\ldots +b_{p}x_{p}\right)$$

where *h*(*t*)is the hazard function at time *t*, *h*_0_ is the unspecified baseline hazard, and *x*_1_, *x*_2_, …*x*_*p*_ are covariates (i.e., biomarker concentrations). The coefficients *b*_1_, *b*_2_, …*b*_*p*_ represent the log hazard associated with a unit change in each covariate [66]. Although the variable *t* traditionally denotes survival time, here it was used in a non-time-dependent context to allow derivation of hazard-like metrics for stratifying patients based on biomarker data alone.

To accommodate the small sample size, presence of biomarker outliers, and a lack of follow up data, the analysis employed conceptual and methodological guidance from several extensions of the Cox model. Weighted Cox Regression was considered to improve interpretation in the presence of non-proportional effects [50], and robust estimation techniques such as trimming were noted for their ability to reduce the influence of extreme values [51]. Additionally, methods incorporating sampling weights into robust Cox estimation were reviewed for relevance to stratified or case-cohort designs [52]. These methodological innovations support the adapted application of the model in this study to generate Xbeta-based risk scores for further classification.

Hazard ratio, the exponentiation of the B coefficient (Exp(B)) were calculated for each biomarker, reflecting multiplicative change in predicted risk per unit increase in concentration. Ratios greater than one indicate increased risk, while values below one indicate a protective or inverse relationship. A hazard ratio of one implies no effect. The 95 % confidence intervals were used to assess the precision of estimates, with significance determined by whether the interval excluded 1 [67].

### 2.5 Risk mitigation matrix

The risk mitigation matrix was developed to categorise individuals into four risk levels (low, medium-low, medium-high, high) based on Xbeta values (linear predictors) derived from Cox Regression. Risk estimation followed the principles of the Heuristic Ideation Technique (HIT), which systematically combines probability and consequence to rank risk severity [54], 58], 68]. In this study, probability was operationalised by biomarker threshold exceedance (IL-6, TNF-α, MPO), while consequence reflected clinical implications associated with risk tier [55], 57].

Individuals were classified according to the number of biomarker thresholds exceeded: one biomarker elevation indicated low risk, two elevation indicated medium-low risk, three elevations indicated medium-high risk and four-five elevations indicated high risk. In this framework, the maximum theoretical HIT was six, as each biomarker (IL-6, TNF-α, MPO) could contribute two points (serum and plasma), although the highest observed score in this dataset was five. This four-tier framework enabled stratification of both patients and volunteers, the latter representing asymptomatic healthy donors with no clinical diagnosis of OA. The analysis was performed cross-sectionally, reflecting current risk inferred from biomarker profiles rather than longitudinal follow-up.

## 3 Results

### 3.1 Biomarker analysis

IL-6, TNF-α, and MPO concentrations were analysed in plasma and serum samples, with significant differences observed between the two sample types. The analysis revealed statistically significant relationships between biomarker concentrations and OA risk. Serum samples yielded higher biomarker concentrations than plasma, particularly for IL-6 and TNF-α, indicating a greater sensitivity for detecting OA-related inflammatory activity. MPO concentrations were comparable between plasma and serum, with serum levels slightly elevated overall. Although absolute concentrations differed, serum and plasma levels were correlated (*p* > 0.05) [63], indicating that matrix selection did not alter stratification or the thresholds presented in Table 1. Variability in absolute values between sample type has been widely reported, with serum generally yielded higher concentrations of IL-6 and TNF-α, and plasma providing greater stability for MPO [40], [41], [42], [43], [44]. This reinforced the value of systemic approaches such as Cox Regression for integrating biomarker data into risk stratification models. All 86 subjects (58 patients and 28 volunteers) contributed paired serum and plasma samples, with validation performed internally using Cox Regression and DFA cross-validation.

**Table 1:** Validation of the Cox regression model for combined, patient-only and volunteer-only data. Metrics include regression coefficients (b), hazard ratios (Exp(b)), 95 % confidence intervals (CI), and chi-square statistics. Negative coefficients indicate reduced survival probabilities with increasing biomarker levels.

Biomarker	Dataset	Regression coefficient (b)	SE	Wald	*p*-value	Hazard ratio (Exp(B))	95 % CI for Exp(B)	Chi-square	*p*-value (omnibus test)
IL-6	Combined	−5.856	0.813	51.938	<0.001	0.003	[0.001–0.014]	55.056	<0.001
	Patients	−5.718	0.983	33.872	<0.001	0.003	[0.000–0.023]	166.048	<0.001
	Volunteers	−5.767	1.327	18.894	<0.001	0.003	[0.000–0.042]	81.564	<0.001
TNF-α	Combined	−7.005	0.896	60.354	<0.001	0.001	[0.000–0.005]	97.257	<0.001
	Patients	−7.154	1.143	39.176	<0.001	0.001	[0.000–0.007]	206.800	<0.001
	Volunteers	−6.958	1.613	18.604	<0.001	0.001	[0.000–0.022]	94.180	<0.001
MPO	Combined	−5.582	0.772	71.672	<0.001	0.000	[0.000–0.000]	207.371	<0.001
	Patients	−11.368	1.751	42.163	<0.001	0.000	[0.000–0.000]	249.908	<0.001
	Volunteers	−8.748	2.100	17.344	<0.001	0.000	[0.000–0.010]	102.523	<0.001

**Figure 1:**
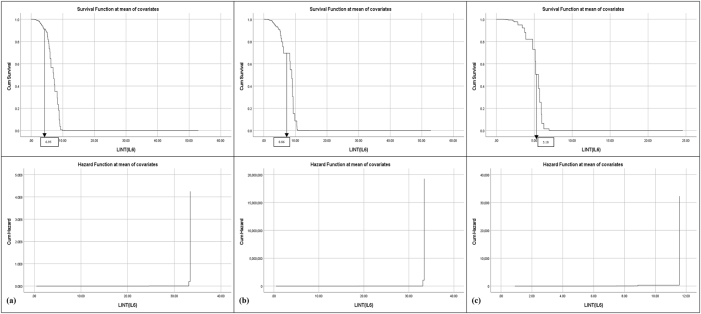
Survival and hazard graphs for IL-6 concentrations derived from Cox regression analysis, displaying (a) combined patient and volunteer data, (b) patient-only data, and (c) volunteer-only data. Threshold values for IL-6 are indicated on each graph.

**Figure 2:**
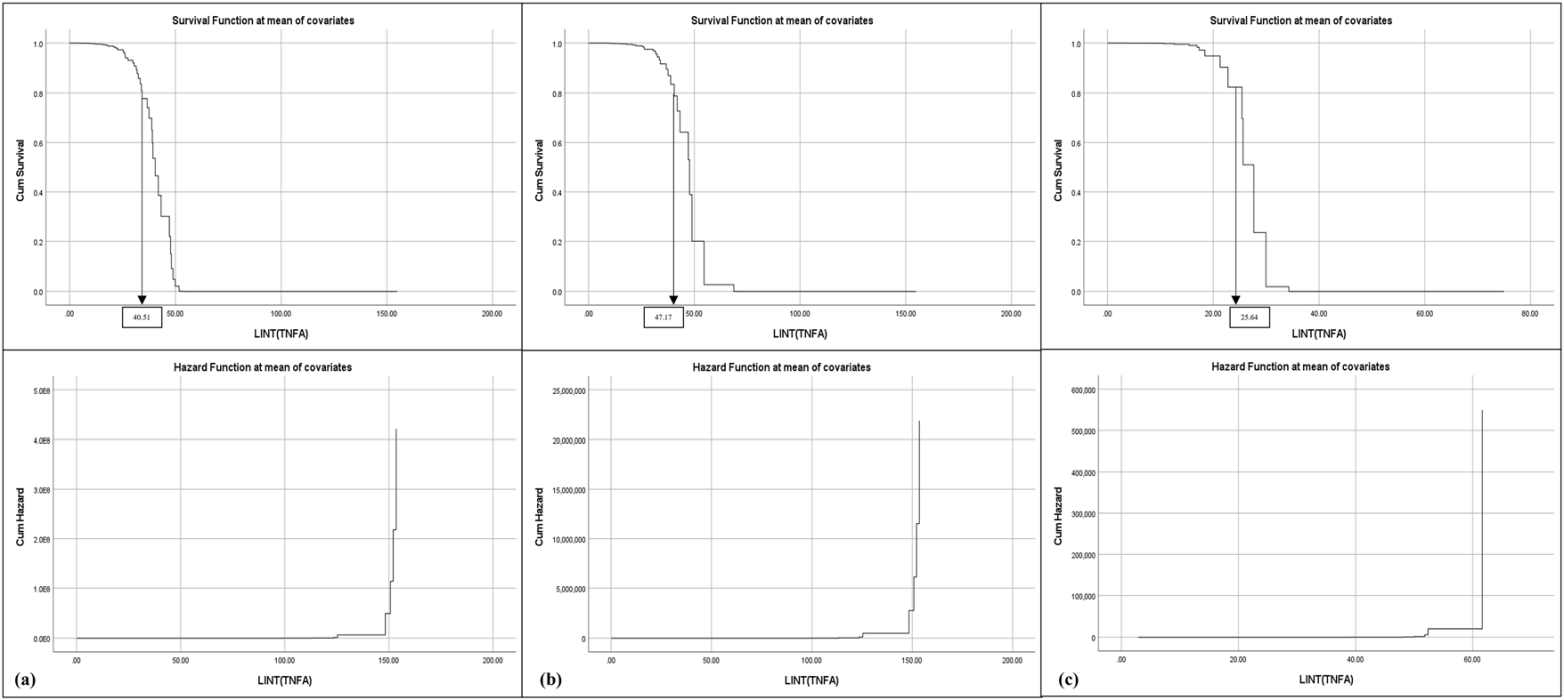
Survival and hazard graphs for TNF-α concentrations derived from Cox regression analysis, displaying (a) combined patient and volunteer data, (b) patient-only data, and (c) volunteer-only data. Threshold values for TNF-α are indicated on each graph.

**Figure 3:**
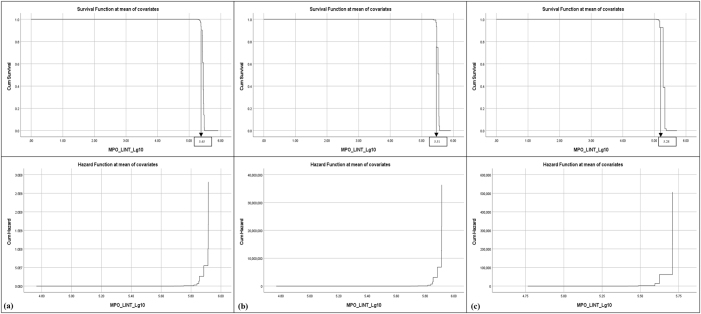
Survival and hazard graphs for MPO concentrations derived from Cox regression analysis, displaying (a) combined patient and volunteer data, (b) patient-only data, and (c) volunteer-only data. Threshold values for MPO are indicated on each graph.

Survival and hazard thresholds for IL-6, TNF-α, and MPO concentrations were shown in Figures 1, 2, and 3. Histograms comparing biomarker distributions across sample types, relative to the 50 % survival threshold, are provided in Supplementary Figures S1–S3. These histograms highlighted variations in plasma and serum biomarker concentrations across patients and volunteers. Survival analysis determined threshold values for OA risk, establishing survival cut offs, for example, IL-6 concentrations ≥ 6.95 pg/mL, TNF-α ≥ 40.51 pg/mL, and MPO ≥ 5.45 pg/mL were indicative of early-stage OA, while higher thresholds marked advanced disease risk [69], 70]. The hazard ratios for these biomarkers were significant, with *p*-values < 0.001, indicating strong associations with OA progression (Table 1). These finding supported the hypothesis that progressive increases in biomarker concentrations correspond with higher OA risk and more severe disease progression.

### 3.2 Comparative analysis

Significant differences were observed in biomarker levels between different subgroups, particularly in relation to age and comorbidities. For instance, older participants (age > 65) had significantly higher IL-6 levels compared to younger participants (*p* = 0.02). Patients with comorbidities such as hypertension and hypercholesterolemia exhibited elevated biomarker levels, suggesting a potential link between these conditions and OA progression [69]. Differences in biomarker thresholds between patients and volunteers further illustrated the impact of disease severity. Patients demonstrated higher thresholds for IL-6 (≥8.86 pg/mL), TNF-α (≥47.17 pg/mL), and MPO (≥5.51 pg/mL) (Table 1). Conversely, volunteers exhibited lower thresholds for IL-6 (≥5.18 pg/mL), TNF-α (25.64 pg/mL), and MPO (5.28 pg/mL), indicating a lower inflammatory burden (Table 1). These variations suggested that biomarker-based risk stratification can effectively differentiate between early and advanced OA.

### 3.3 Cox Regression

Cox Regression analysis evaluated the predictive power of IL-6, TNF-α, and MPO for OA progression, generating survival and hazard thresholds for each biomarker. Survival thresholds for IL-6, TNF-α, and MPO were identified at 6.95 pg/mL, 40.51 pg/mL, and 5.45 pg/mL, respectively. Individuals with biomarker concentrations exceeding these survival thresholds exhibited significantly lower survival probabilities (Figures 1, 2, and 3).

In addition to survival thresholds, hazard thresholds were identified as 30 pg/mL (IL-6), 125 pg/mL (TNF-α), and 5.8 pg/mL (MPO). These thresholds along with the Regression coefficients, hazard ratios, and statistical metrics, are detailed in Table 1 (combined data, patient-only data, volunteer-only data). Validation of the model through chi-square testing confirmed its statistical robustness, with MPO showing the highest predictive value for OA progression (chi-square = 207.371, *p *< 0.001).

The survival and hazard graphs (Figures 1, 2, and 3) and the data presented in Table 1 collectively demonstrate the strong inverse relationships between biomarker levels and survival probabilities. These results support the use of IL-6, TNF-α, and MPO as predictive markers for OA progression, with hazard thresholds providing critical insights for clinical stratification of risk.

### 3.4 Risk mitigation matrix

**Figure 4:**
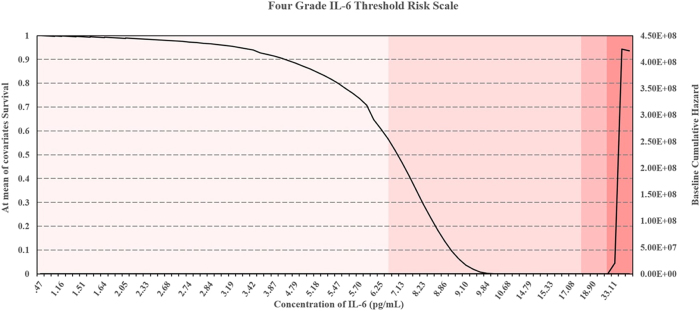
Four-grade threshold risk scale for IL-6 concentrations, stratifying risk levels into low, low/medium, medium/high, and high based on survival and hazard thresholds.

**Figure 5:**
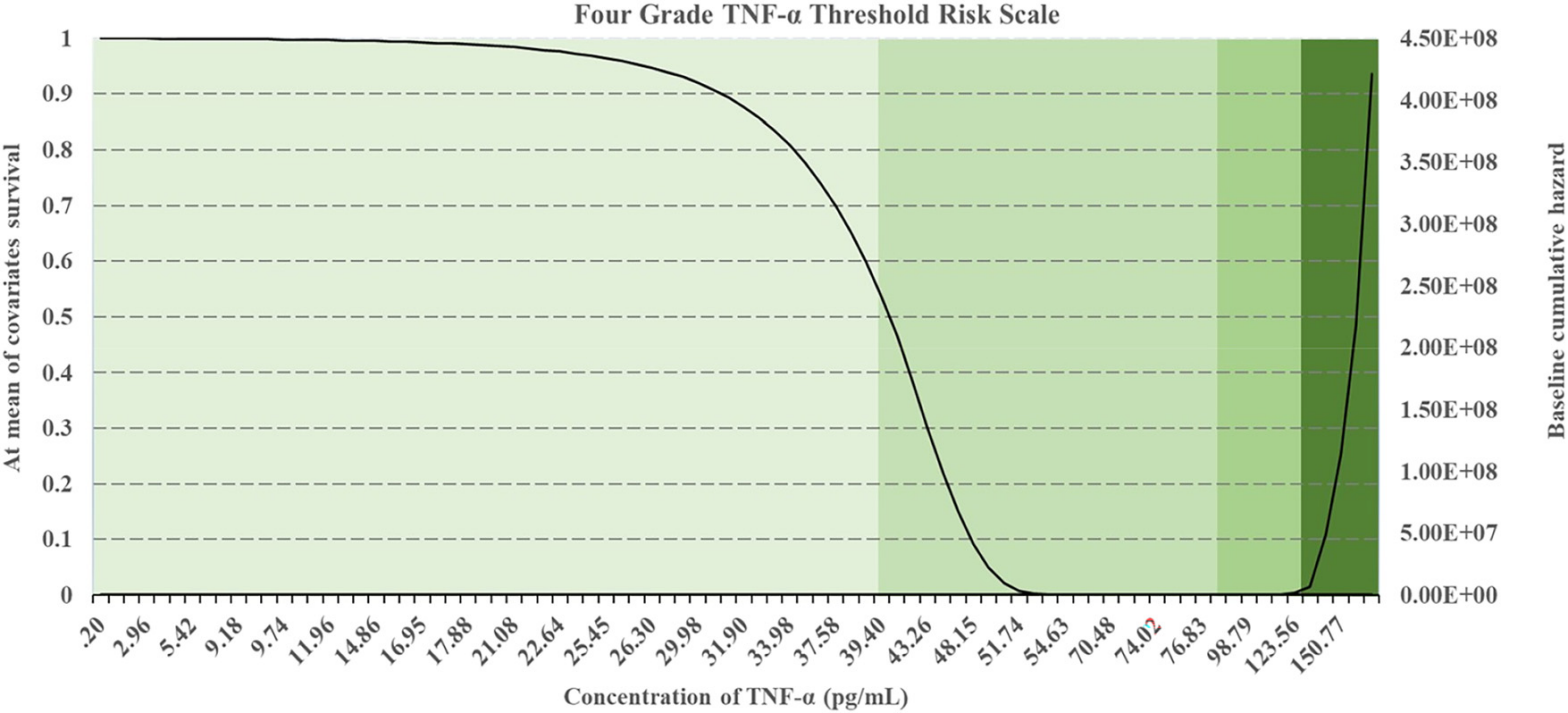
Four-grade threshold risk scale for TNF-α concentrations, stratifying risk levels into low, low/medium, medium/high, and high based on survival and hazard thresholds.

**Figure 6:**
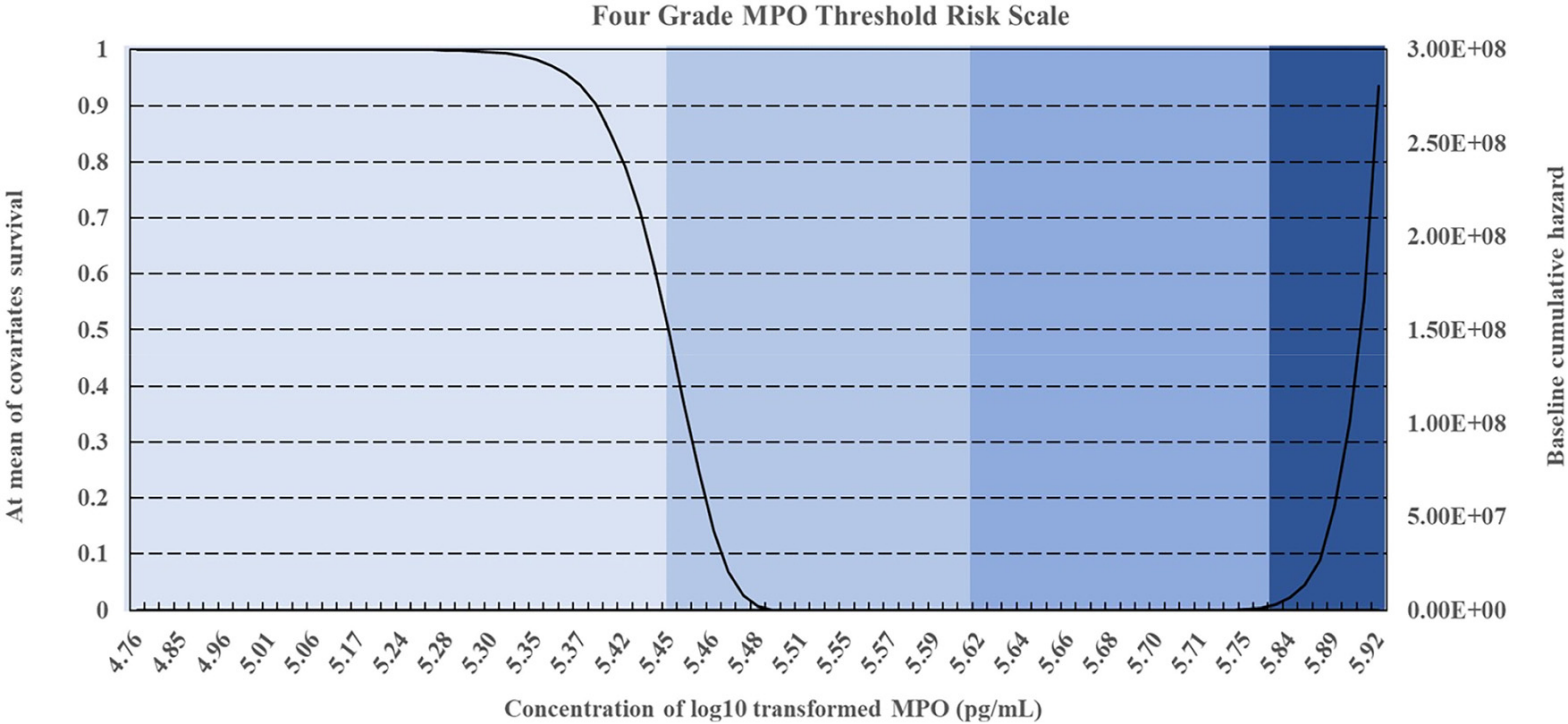
Four-grade threshold risk scale for MPO concentrations, stratifying risk levels into low, low/medium, medium/high, and high based on survival and hazard thresholds.

Patients of interest were identified based on Xbeta values derived from Cox Regression analysis, with the risk mitigation matrix ranking individuals by OA risk. The matrix categorised patients and volunteers into four risk levels, high, high-medium, medium-low, low (Table 2). Patients with multiple elevated biomarkers were classed as high risk, while those with only one or two biomarker elevations were categorised as low or moderate risk. The corresponding four-grade threshold risk scales for IL-6, TNF-α, and MPO were shown in Figures 4, 5, and 6. These scales provided a comprehensive framework for assessing biomarker-driven risk stratification.

**Table 2:** Risk mitigation matrix categorising patients and volunteers by biomarker concentrations (IL-6, TNF-α, MPO) and associated risk levels derived from Cox regression analysis. HIT (Heuristic Ideation Technique) indicates the number of elevated biomarkers. Plasma (P) and serum (S) concentrations are shown, with risk stratified as high, high/medium, medium/low, or low based on survival and hazard thresholds.

Study ID	HIT	Level of risk	IL-6 (pg/mL)	TNF-α (pg/mL)	MPO (pg/mL)
P30	5	High	15.33 (S)	101.94 (S)	5.66 (S)
P26	5	High	14.79 (S)	123.56 (S)	5.92 (P)
P27	4	High/Medium	20.44 (S)	154.84 (S)	5.92 (P)
P23	4	High/Medium	10.51 (P)	85.78 (S)	5.56 (S)
P25	4	High/Medium	9.84 (S)	72.93 (P)	5.57 (S)
P32	4	High/Medium	18.10 (S)	148.33 (P)	5.71 (P)
P20	3	Medium	11.55 (P)	72.48 (P)	5.59 (S)
P18	3	Medium	8.61 (S)	85.78 (S)	5.48 (P)
P29	3	Medium	33.11 (P)	25.31 (P)	5.30 (P)
V15	3	Medium	18.90 (P)	7.54 (P)	5.71 (P)
P31	3	Medium	14.88 (S)	11.98 (S)	5.45 (S)
P17	2	Medium/Low	52.90 (S)	33.47 (S)	5.55 (S)
P19	2	Medium/Low	3.72 (P)	98.79 (S)	5.92 (P)
P28	2	Medium/Low	5.30 (P)	148.33 (P)	5.46 (S)
P15	2	Medium/Low	18.10 (S)	85.78 (S)	5.56 (S)
P35	1	Low	5.47 (P)	10.64 (P)	5.84 (P)
V10	1	Low	4.65 (P)	15.86 (P)	5.54 (P)
V2	1	Low	3.33 (P)	26.22 (S)	5.48 (P)

The risk mitigation matrix informed intervention strategies, patients at lower risk could benefit from lifestyle modifications and early medication, while those at moderate risk might require physiotherapy and increased medication doses. High-risk patients were more likely to need surgical interventions, such as total joint replacement. Xbeta graphs highlighting patients and volunteers of interest are provided in Supplementary Figures S4–S6.

The matrix also evaluated patient responses to identified hazards over time, refining risk trajectories [55], 57]. Cross-validation with the four-grade threshold scale confirmed its reliability. The thresholds and stratifications presented in Tables 1 and 2 further validate the utility of this this framework for clinical decision-making. Future research should further validate this approach with larger cohorts and explore additional biomarkers to enhance predictive accuracy.

### 3.5 Integration and Discriminant Function Analysis and Cox Regression

Discriminant Function Analysis (DFA) and Cox Regression could be used as complementary methodologies to enhance risk stratification. DFA classified participants into risk categories based on biomarker concentrations, achieving a classification accuracy of 71.8 % [71]. However, its deterministic nature limited its applicability for modeling time-dependent risk. Cox Regression addressed this limitation by incorporating temporal assessment, refining survival and hazard thresholds for IL-6, TNF-α, and MPO.

This integration of these methodologies, validated through the risk mitigation table strengthens the reliability of the risk stratification model by combing categorical classification with survival-based risk estimation (Table 2).

## 4 Discussion

### 4.1 Key findings

The analysis revealed statistically significant relationships between biomarker concentrations and OA risk, with IL-6 [X^2^ [71] = 268.287, *p* < 0.001], TNF-α [X^2^ [71] = 319.823, *p* < 0.001] and MPO [X^2^ [71] = 422.943, *p* < 0.001] showing highly significant negative regression coefficients (Table 1, Figures 1, 2, and 3) [48], 70]. These findings indicate strong inverse associations between biomarker levels and survival probabilities, reinforcing their predictive value in distinguishing between early and advanced OA stages [69].

Threshold concentrations derived from the analysis serve as practical benchmarks, with IL-6 concentrations ≥ 6.95 pg/mL, TNF-α ≥ 40.51 pg/mL, and MPO ≥ 5.45 pg/mL indicative of early-stage OA, and higher thresholds marking advanced disease with potential comorbidities (Figures 4, 5, and 6) [33], 72]. Comparisons between patient and volunteer subgroups demonstrated that patients exhibited higher threshold values (e.g., IL-6 ≥ 8.86 pg/mL, Table 1, Figure 4), reflecting inflammatory and oxidative stress burdens associated with OA progression. In contrast, volunteers displayed lower thresholds (e.g., IL-6 ≥ 5.18 pg/mL, Table 1, Figure 4), indicative of a reduced inflammatory burden. The risk stratification approach confirmed that higher biomarker concentrations correlated with survival probabilities, supporting their role in identifying disease severity.

The integration of DFA with Cox Regression further validated the predictive capability of these biomarkers (Supplementary Figures S4–S6). While DFA classified individuals based on biomarker concentrations, Cox Regression refined disease progression risk estimates by incorporating survival analysis. Cross validation between the four-grade threshold scale (Figures 4, 5, and 6) and the risk mitigation matrix (Table 2) confirmed their consistency, reinforcing their clinical applicability. While assays were carefully standardised, methodological variability across platforms remains a recognised challenge for clinical translation. These considerations highlight the needs for ongoing validation to strengthen the generalisability of the derived threshold.

Previous OA research has shown the value of Cox Regression for assessing lifestyle and demographic influence, for example diet [49], 73], 74], exercise [75] and age related cytokine profiles [26]. These studies highlight important determinants in OA risk, but they have not typically provided reproducible biomarker thresholds. The represent study complements this work by applying Cox Regression and DFA to derive quantitative cut-offs for IL-6, TNF-α, and MPO and embedding them within a four-stage risk rating system and risk mitigation matrix. This framework builds a foundation for integrating biomarkers with lifestyle, demographic, and clinical factors in future longitudinal studies, advancing toward comprehensive multimodal tools for OA prognosis.

### 4.2 Clinical implications

The risk mitigation matrix (Table 2) and four-grade threshold scale (Figures 4, 5, and 6) developed in this study provided practical tools for clinicians to stratify OA risk and guide treatment decisions. By integrating these tools into routine clinical practice, clinicians could achieve more accurate staging and prognosis of OA. Patients with high IL-6 levels (Figure 4) may benefit from anti-inflammatory treatments, while those with high TNF-α levels (Figure 5) might require different therapeutic approaches targeting specific inflammatory pathways [76], 77].

By adapting Cox Regression to accommodate biomarker concentrations rather than traditional time-to-event data, the study provided a practical framework for cross-sectional datasets, particularly in settings where longitudinal follow-up was not feasible. Unlike radiographic classification systems such as the Kellgren–Lawrence (KL) scale, which primarily detect structural changes in moderate to late disease stages [14], 60], 61], 78], 79], the biomarker-based framework offers earlier prognostic insight by quantifying inflammatory and oxidative stress activity [7]. This distinction supported its clinical applicability as a complementary tool, enabling risk stratification before irreversible joint damage occurs.

The four-grade threshold scale and risk mitigation matrix derived here were conceptually aligned with KL stages to illustrate how they could support integrated clinical decision-making. For low-risk individuals (comparable to KL grades 0–1) may be managed with preventive lifestyle interventions such as exercise, diet modification, and cardiovascular risk monitoring. Survival models have demonstrated that dietary improvements [49], 73], 74] and structured exercise interventions [75] reduce OA progression risk, underscoring the relevance of early behavioural modification. In medium-low risk patients (KL grade 2) could benefit from physiotherapy, weight management, and non-steroidal anti-inflammatory drugs (NSAIDs) to alleviate symptoms and slow structural progression [63], [80], [81], [82]. Medium-high risk groups (KL grade 3) may require more aggressive pharmacological interventions, including disease-modifying agents currently under clinical evaluation [34], 35], 83]. Finally, high-risk individuals (KL grade 4), particularly those exceeding multiple biomarker thresholds, are more likely to require surgical evaluation and early consideration of joint replacement [84], [85], [86].

When used alongside imaging assessments, this biomarker-driven system would provide a more integrated view of disease activity and prognosis. KL grading and MRI [87] remain critical for assessing structural severity [14], 60], 61], 78], 79], but they are limited by insensitivity to early biochemical changes, inter-observer variability, and weak correlation with symptoms [88]. Biomarker thresholds, by contrast, reflect biological activity and can identify patients at risk of progression before radiographic features become apparent. Thus, the two systems should be regarded as complementary: radiographic tools capture structural damage, while biomarker-based models measure dynamic inflammatory burden and predict risk trajectories. Incorporating both perspectives may refine staging, personalise treatment strategies, and ultimately improve outcomes in patients with OA [89].

The matrix categorised patients into high, high-medium, medium-low, low risk groups, facilitating personalised treatment strategies. Medium-risk patients may benefit from physiotherapy and pharmacological management, while high-risk individuals with multiple elevated biomarkers may require early surgical intervention. Integrating biomarker-based stratification with imaging modalities such as MRI or X-ray could further enhancing staging accuracy, optimising treatment planning and patient outcomes.

### 4.3 Future research

Prognostic modelling in OA carries inherent challenges, including modest sample sizes, variability in assay methods, and the influence of lifestyle factors. These were considered in this study and provide direction for future research priorities.

Future research should validate these biomarker thresholds in larger, multi-centre cohorts to enhance generalisability. Additionally biomarkers, such as C-reactive protein and matrix metalloproteinases, should be investigated to further refine the predictive accuracy of the proposed tools [90]. Additionally, incorporating clinical and lifestyle factors such as exercise, BMI, diet, blood pressure, and cholesterol into the analysis could provide a more comprehensive understanding of OA risk factors [91], 92].

Importantly, longitudinal studies are required to track biomarker changes alongside symptom improvement or disease progression, enabling the development of prediction models that reflect real-time clinical outcomes. Such approaches would extend the present cross-sectional framework into a tool for monitoring treatment response and tailoring management strategies. This is particularly relevant as IL-6, TNF-α, and MPO map onto inflammatory and oxidative stress pathways that overlap with targets of emerging DMOADs (e.g., IL-1 inhibitors, RORα antagonists, FGF-18, BMP-7), suggesting these biomarkers may serve not only as prognostic indicators but also as surrogate markers for therapeutic efficacy [34], [35], [36], [37].

Recent studies have demonstrated the impact of dietary factors on OA progression using Cox Regression analyses [73], 74] and the effectiveness of exercise interventions for OA patients [75], highlighting the importance of these variables in future research. Longitudinal studies are needed to assess biomarker stability and predictive value for OA progression. Integrating biomarkers with machine learning and imaging could refine diagnostics and treatment advancing precision medicine to improve outcomes and reduce OA-related healthcare burdens.

Further exploration of alternative Cox Regression methods, such as weighted or robust models [50], [51], [52] could enhance the model’s applicability across diverse clinical settings, especially in datasets with outliers or design limitations. Incorporating tissue culture models and synthetic data could expand the dataset, validate findings and confirm the robustness of this approach. These enhancements could improve the utility of the risk mitigation matrix (Table 2) and the survival and hazard thresholds (Table 1) as tools for personalised OA management.

## 5 Conclusions

This study demonstrated the utility of adapted Cox Regression model, replacing traditional time-to-event variables with biomarker concentrations, to evaluate predictive thresholds for OA risk [93]. This methodological adjustment, while non-traditional, was supported by conceptual advances in Cox modelling that addresses challenges in observational design, outlier influence, and model flexibility [50], [51], [52]. By quantifying IL-6, TNF-α, and MPO concentrations, this study provided a standardised, reproducible approach to biomarker interpretation in OA prognosis [54], 70].

The development of a risk mitigation matrix (Table 2) and four-grade threshold scale offers practical tools for clinicians, enhancing the early detection and management of OA [55], 90]. Validation of the Cox Regression model across combined, patient-only and volunteer-only datasets (Table 1) ensures resilient threshold estimates and highlights the clinical relevance of these biomarkers. The integration of Cox Regression with Discriminant Function Analysis further strengthens the accuracy of risk stratification, supporting the clinical applicability of biomarker-based models [71].

By incorporating these biomarkers into a structured risk framework, this study establishes predictive thresholds for OA progression, advancing the understanding of OA and offering tools for early diagnosis and intervention [73], 74]. Future research should focus on standardising these tools for clinical use, integrating them with imaging and other diagnostic modalities, and addressing potential challenges related to data collection and variability [39].

## Supplementary Material

Supplementary Material
