# Supplementary material for: Production of a risk mitigation matrix and four-grade threshold scale using Cox Regression to predict osteoarthritis development
Source: Open Life Sci. 2026 Jan 13;21(1):20251193. doi: 10.1515/biol-2025-1193 (PMC12917557; doi:10.1515/biol-2025-1193)
Supplement: Supplementary file 1 — Supplementary Material [file j_biol-2025-1193_suppl_001.docx]

**Supplementary Material Captions**

**
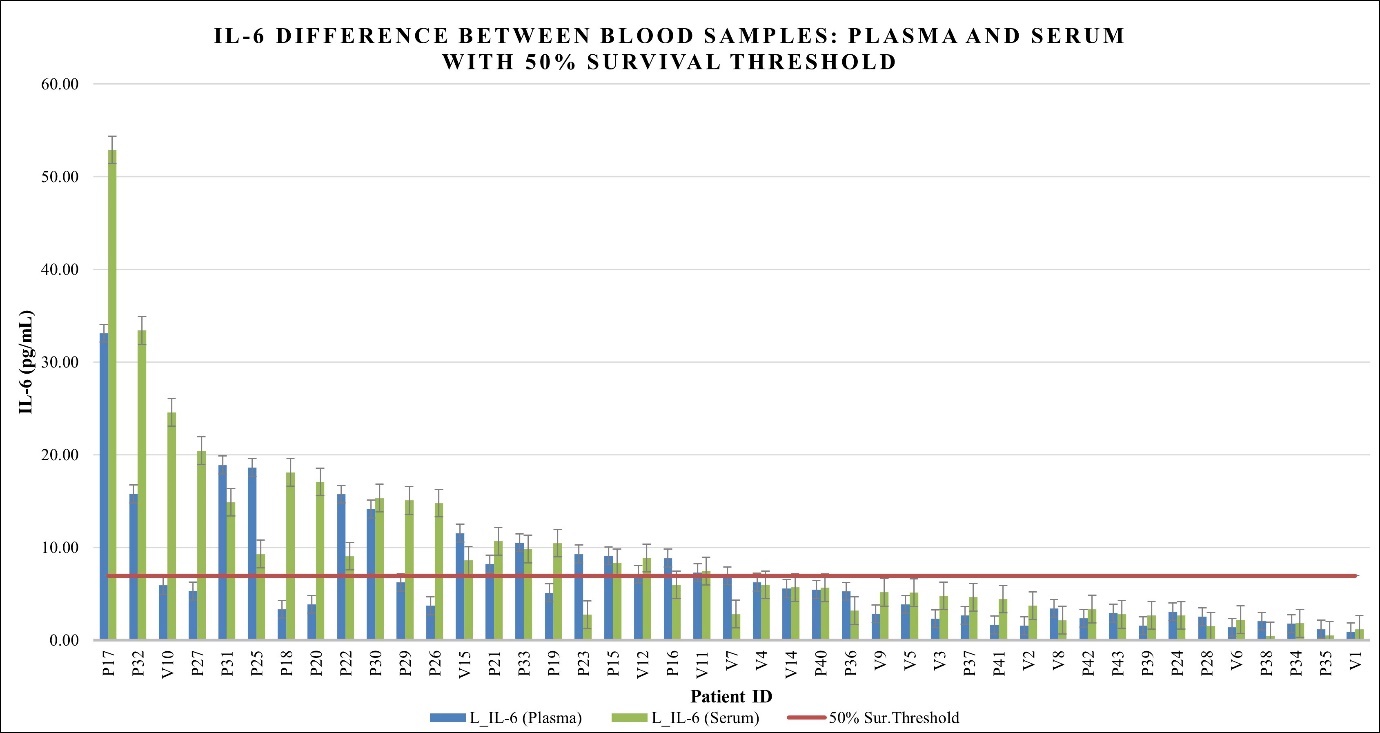
**

**Figure S1.** IL-6 concentrations in plasma and serum samples, showing differences across patients and volunteers with the 50% survival threshold indicated by the red line.


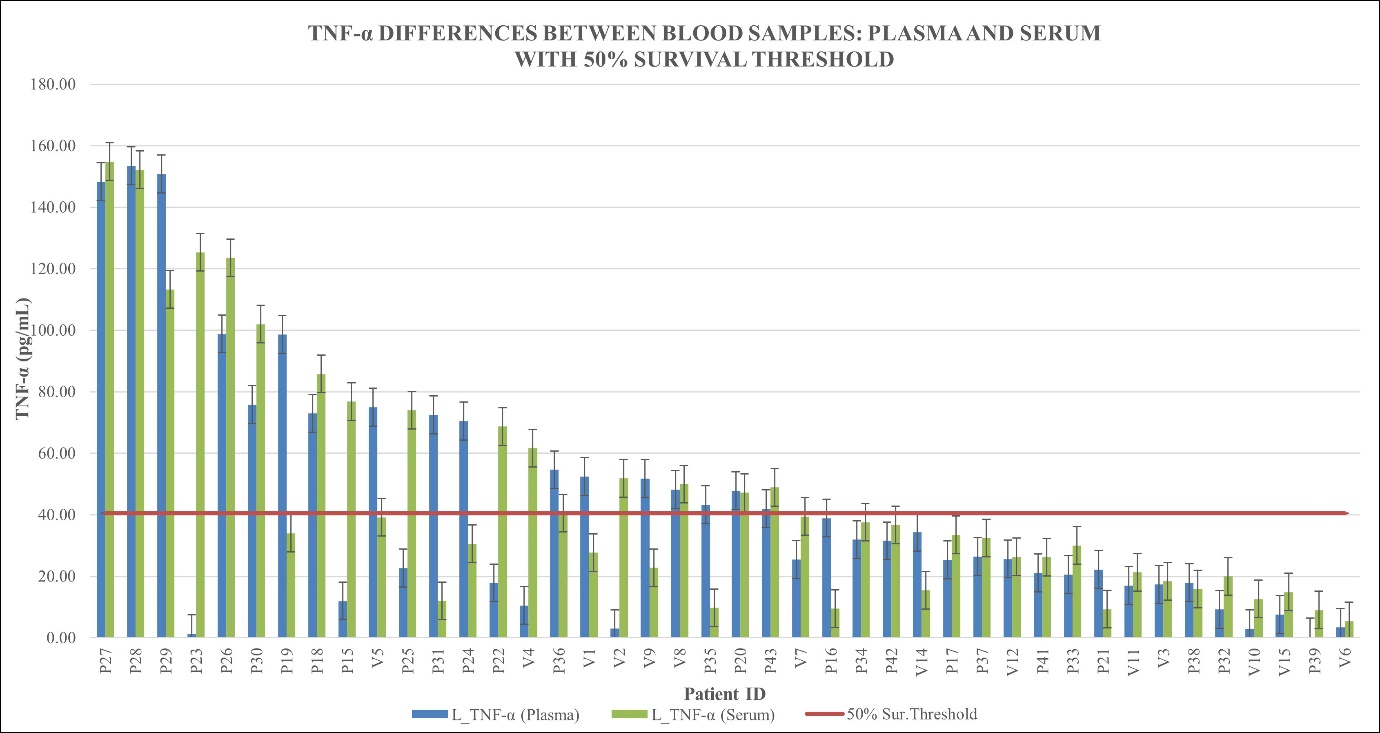


**Figure S2.** TNF-α concentrations in plasma and serum samples, showing differences across patients and volunteers with the 50% survival threshold indicated by the red line.


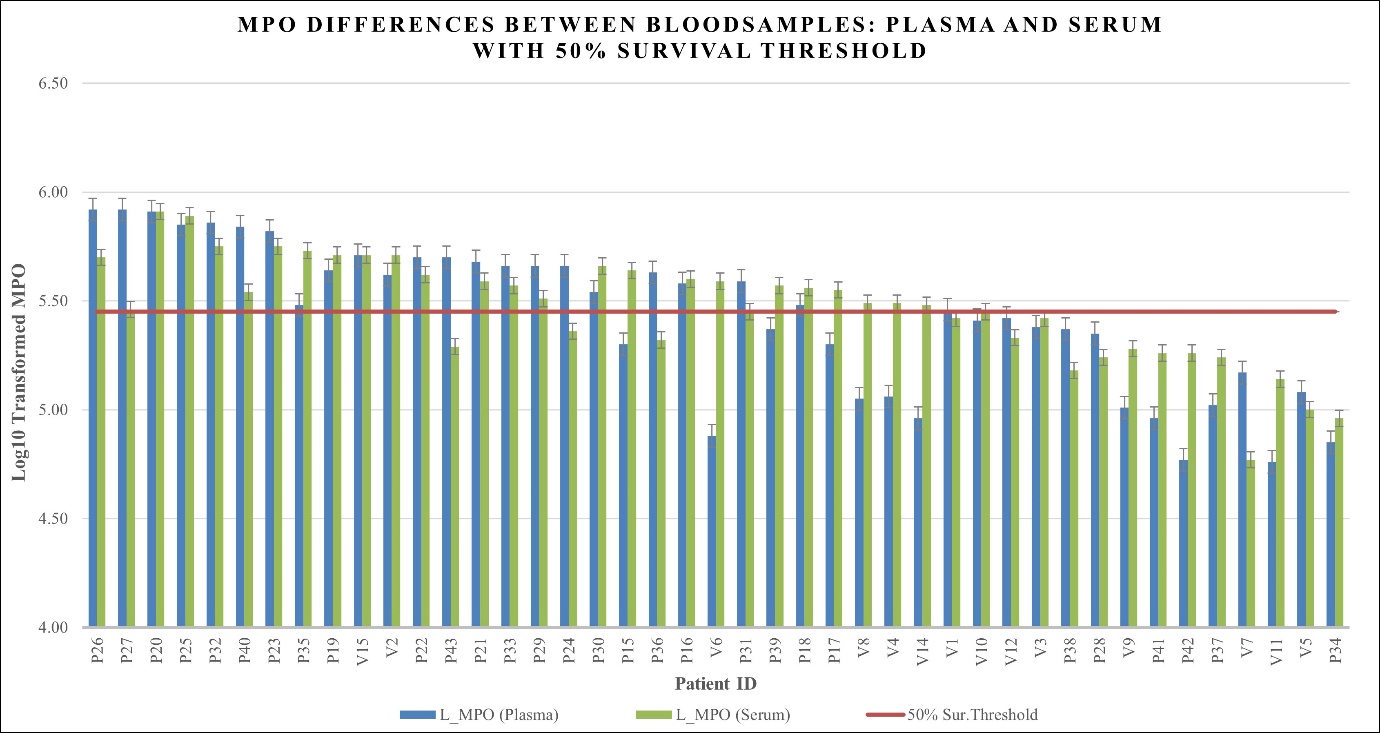


**Figure S3.** MPO concentrations in plasma and serum samples, showing differences across patients and volunteers with the 50% survival threshold indicated by the red line.


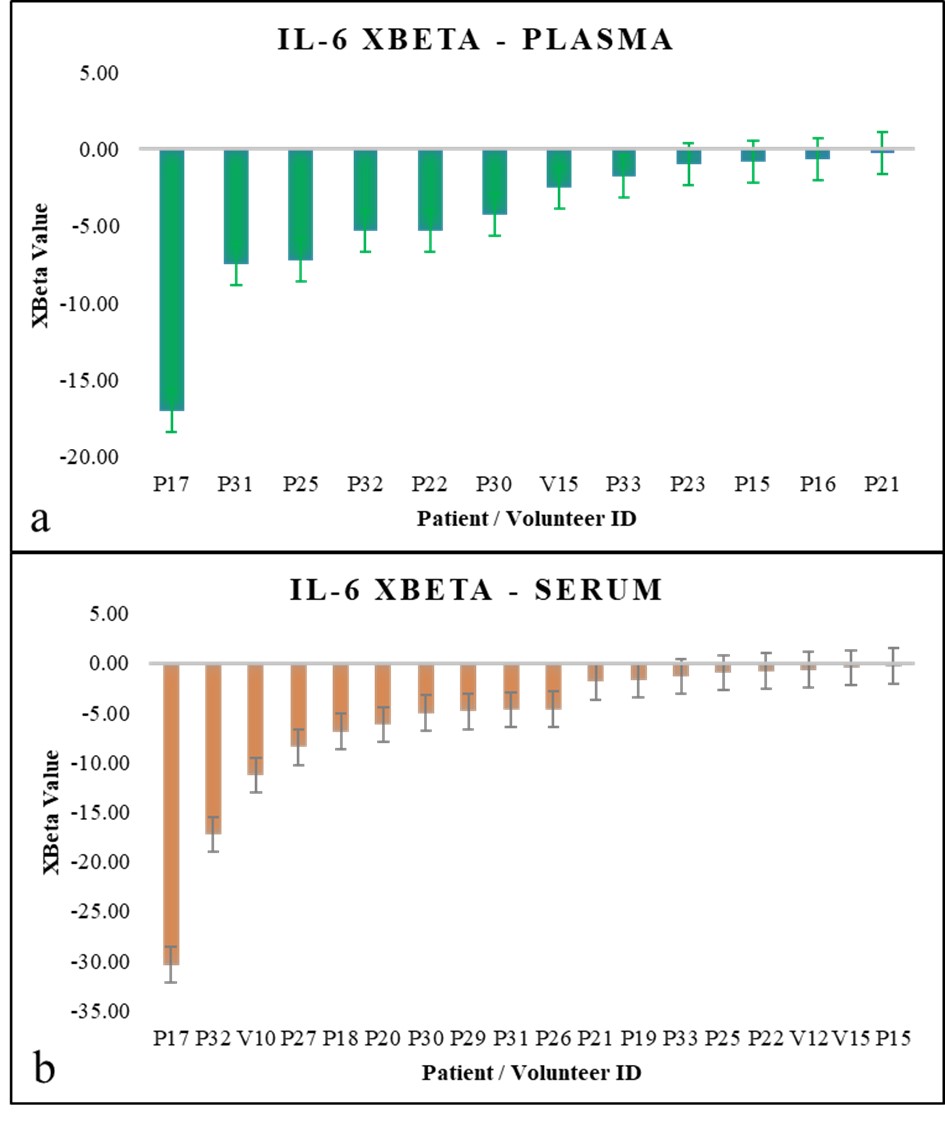


**Figure S4.** Xbeta graphs for IL-6 concentrations in (a) plasma and (b) serum samples, highlighting patients and volunteers of interest (*) based on Cox regression analysis.


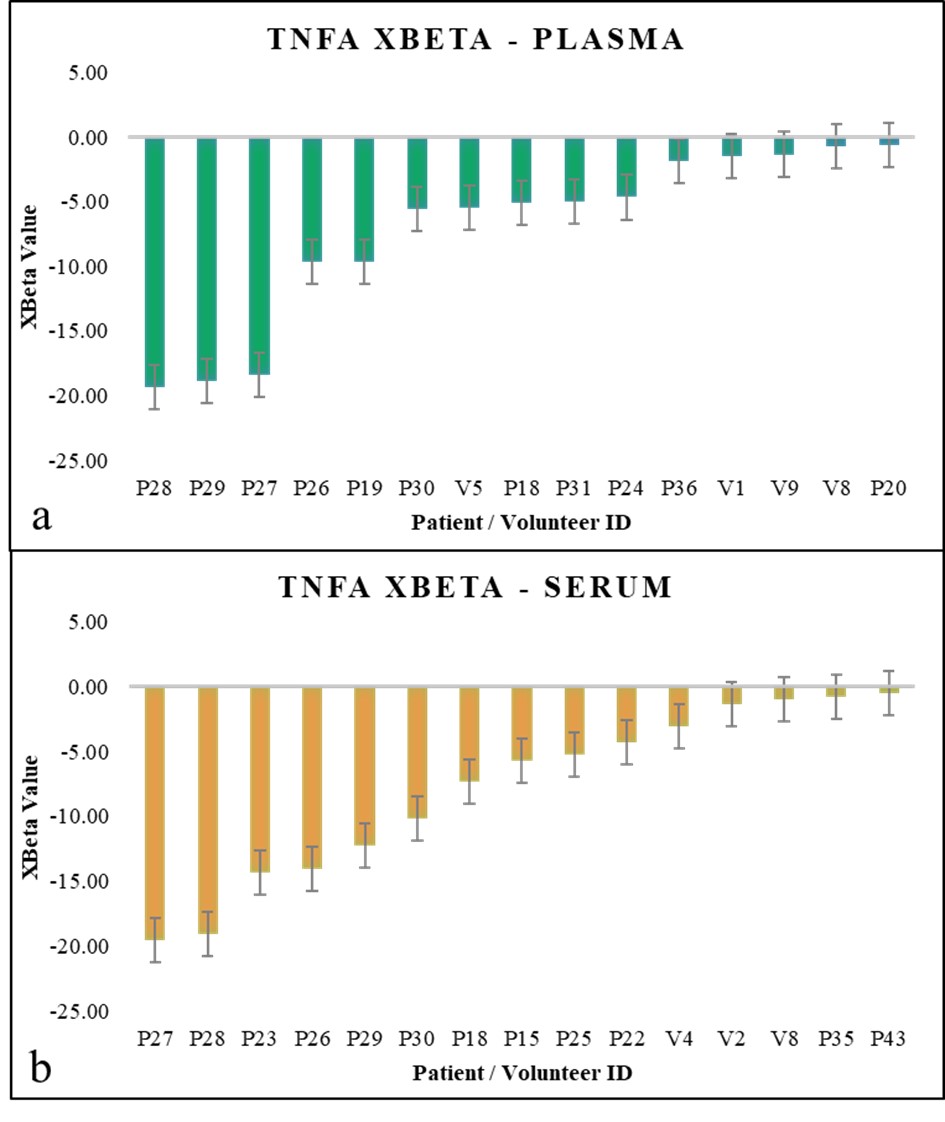


**Figure S5.** Xbeta graphs for TNF-α concentrations in (a) plasma and (b) serum samples, highlighting patients and volunteers of interest (*) based on Cox regression analysis.


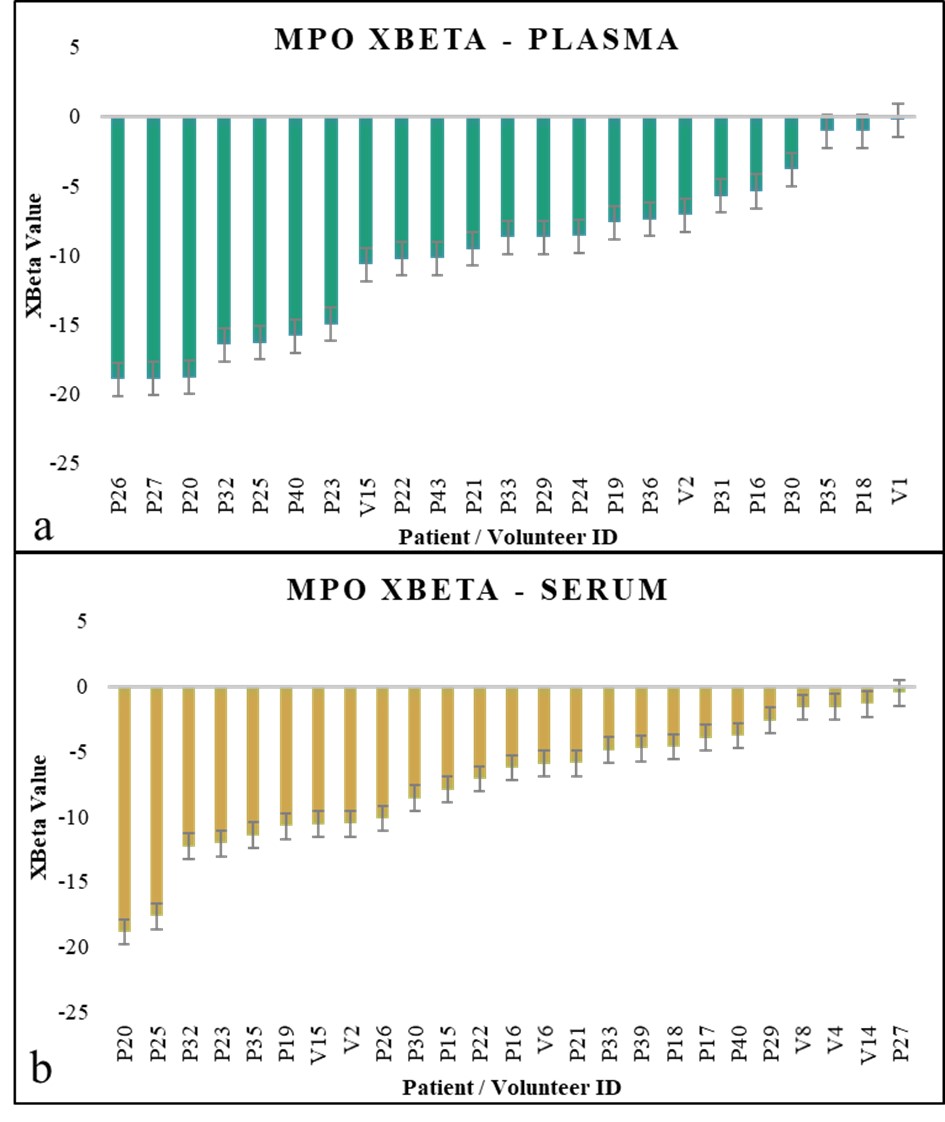


**Figure S6.** Xbeta graphs for MPO concentrations in (a) plasma and (b) serum samples, highlighting patients and volunteers of interest (*) based on Cox regression analysis.
